# Metastases to the Kidney: An Analysis of 35 Cases and a Review of Literature

**DOI:** 10.3389/fonc.2020.632221

**Published:** 2021-02-19

**Authors:** Jinchao Chen, Nienie Qi, Shaoxing Zhu

**Affiliations:** ^1^ Department of Urologic Surgery, The Cancer Hospital of the University of Chinese Academy of Sciences/Zhejiang Cancer Hospital, Institute of Basic Medicine and Cancer (IBMC), Chinese Academy of Sciences, Hangzhou, China; ^2^ Department of Urology, The Affiliated Hospital of Xuzhou Medical University, Xuzhou, China

**Keywords:** kidney, metastasis, diagnosis, treatment, prognosis

## Abstract

**Introduction:**

In addition to being rare, metastases to the kidney present clinicians with issues regarding their treatment.

**Materials and Methods:**

We retrospectively analyzed 35 cases of diagnosed renal metastases. The clinical characteristics, imaging features, pathological features, diagnosis, and treatment were analyzed, and Kaplan-Meier methods and Cox regression analysis were used to calculate overall survival (OS) and influencing factors.

**Results:**

The average age of the patients was 62 years, and 40% presented with symptoms. The most common primary tumor was lung cancer (60%), and two patients had renal metastases coexisting with renal cell carcinoma. The average interval from primary tumor to renal metastasis was 29.4 months. Only 45.5% of the patients who underwent enhanced computerized tomography were diagnosed with renal metastases. Renal biopsy was performed in 16 patients (45.7%), leading to a diagnosis in 15 (93.8%). Twenty-one patients (60%) received surgical treatment, and median recurrence free survival of these patients was 7 months (95% CI, 5 to 12). Overall, the median OS was 44 months for patients who underwent renal surgery, and 52 months for patients who did not (P = 0.672). However, for patients without metastases at other sites, surgery could significantly prolong OS (P = 0.001).

**Conclusion:**

Although rare, the possibility of renal metastasis should be considered after finding renal tumors in patients with primary tumors in other organs, and can be diagnosed by imaging examination and puncture biopsy. For patients without other metastases, surgical intervention can be considered for the renal lesions.

## Introduction

Renal cell carcinoma (RCC) is the most common malignant kidney tumor; however, metastases to the kidney are relatively rare. In the past, there have been few reports on the incidence of renal metastases, and autopsy results show that the incidence of renal metastases was approximately 2.36–12.6% (1, 2). Most studies on renal metastases were case reports, and there is a lack of systematic description of clinical features, diagnosis, treatment, and disease prognosis.

The most common primary tumors are lung, breast, gastric, and colon cancer, with melanoma also being high on the list (3). Clinical manifestations include flank pain and hematuria, but most cases are asymptomatic, and the tumor is usually found incidentally on imaging examination (4). Diagnosis can be made based on renal biopsy or surgical pathology. Thus far, treating renal metastases has been a perplexing problem for many urologists and oncologists, and surgery remains a controversial approach.

In this study, we analyzed 35 cases of renal metastasis at the Cancer Hospital of the University of Chinese Academy of Sciences and the Affiliated Hospital of Xuzhou Medical University, and tried to describe their clinical features, diagnosis, and treatment methods.

## Materials and Methods

The study was both approved by Medical Ethics Committee of Zhejiang Cancer Hospital and Medical Ethics Committee of the Affiliated Hospital of Xuzhou Medical University. We retrospectively collected a total of 35 cases of renal metastases, of which 32 were diagnosed pathologically at the Cancer Hospital of the University of the Chinese Academy of Sciences and three were diagnosed at the Affiliated Hospital of Xuzhou Medical University from January 2003 to December 2019. The inclusion criteria were as follows: malignant tumors other than kidney were pathologically confirmed, renal metastases were pathologically diagnosed following puncture biopsy or surgical resection, and the source of renal metastases was consistent with the primary lesion. All clinical data were detailed, and all patients with renal metastases had imaging data, including ultrasound, computerized tomography (CT), magnetic resonance imaging (MRI), or positron emission tomography/computerized tomography (PET/CT). Exclusion criteria were direct extension of the tumor to the kidney instead of true metastases to the kidney.

The clinical data of the cases were collected, including demographic information, time of diagnosis of the primary lesion(s), time of occurrence of renal metastases, symptoms, imaging features (B ultrasound, CT, MRI, PET/CT), metastasis at other sites, puncture pathology, operation, and complications. The number, location, size, and enhancement characteristics of the renal lesions were evaluated by a specialized radiologist. The biopsy or surgical specimens were evaluated by pathologists and, if necessary, immunohistochemical staining was performed to confirm the diagnosis. The included cases were followed up to record the survival status, and 34 cases were followed up. The overall survival (OS) of the patients was the time from the diagnosis of the primary lesion to the last follow-up or death.

The χ^2^ test and the t-test were used to compare categorical and continuous variables, respectively. OS was calculated using the Kaplan–Meier method for the study population. The log-rank test was used to compare the survival rates between groups. We also used multivariate Cox regression analysis to explore the factors influencing OS. All statistical analyses were performed using SPSS V.19 statistical software. P values < 0.05 were considered statistically significant.

## Results

The average age of patients was 62.0 years old (45–83 years old) and 65.7% (23/35) of the patients were male. Forty percent of the patients presented with clinical manifestations, such as lower back pain and hematuria, while 60% presented with no obvious symptoms. In 54.3% (19/35) of the cases, the cancer had metastasized to other organs besides the kidneys. Simultaneous primary lesions and renal metastases were found in four patients, with the longest interval between the two being 156 months and the average being 29.4 months (**Table 1**). Regarding the source of the primary tumor, the most common was lung cancer (60%), followed by colorectal (8.6%), esophageal (5.7%), breast (5.7%) and ovarian (5.7%) cancers. Liver cancer, endometrial cancer, thyroid cancer, parotid tumor, and melanoma were all equally present (2.9%).

**Table 1:** Demographics and clinical characteristics.

		Total	Renal surgery	No renal surgery	P value
Number		35	21	14	
Age (mean)		62.0	63.2	60.2	0.41
Gender	Male	23	13	10	0.42
	Female	12	8	4	
Symptoms	Yes	14	8	6	0.53
	No	21	13	8	
Primary organ site	Lung	21	12	9	0.47
	Others	14	9	5	
Other site metastasis	Yes	19	8	11	0.021
	No	16	13	3	
Time to renal metastasis (mean, months)		29.4	30.6	27.7	0.82
Diameter of largest renal mass (mean, cm)		4.4	4.6	4.3	0.71

A total of 94.3% (33/35) of patients underwent enhanced CT, followed by PET/CT (10/35) and MRI (1/35). Eighty percent of cases with renal metastases were unilateral and 85.7% of the patients were solitary. The average maximum tumor diameter was 4.4 cm (1–8.4 cm). Among the 33 patients who underwent enhanced CT scan, only 45.5% were diagnosed with secondary renal tumors and 28.6% were diagnosed with primary renal tumors; it could not be clarified whether the tumor was primary or secondary in the remaining patients. Contrast-enhanced CT showed irregular patchy low-density foci, most of which were endogenous, with unclear boundaries, mild to moderate enhancement, and weaker than normal renal parenchyma.

Renal biopsy was performed in 16 patients (45.7%), of whom 15 (93.8%) were pathologically diagnosed with renal metastases. One patient underwent nephrectomy due to renal hemorrhage after the puncture. There were no cases of needle metastasis after puncture. Twenty-one patients (60%) received surgical treatment; there were no significant differences in sex, age, symptoms, type of primary focus, time of occurrence of renal metastases, size of renal tumor between patients who underwent surgery and those who did not, but there was a significant difference in whether there was another metastatic site (P = 0.021) (**Table 1**). Surgery was performed with curative intent in nine patients (42.9%), for a suspected primary tumor in five patients (23.8%), for palliation in three patients (14.3%), for symptom relief in three patients (14.3%), and for stopping bleeding after renal puncture in one patient (4.8%). Of the patients who underwent surgery, 16 (76.2%) underwent radical nephrectomy and five (23.8%) underwent partial nephrectomy. One patient died of multiple organ failure after surgery. Postoperative pathology revealed that there were two patients with renal metastases complicated by primary renal cell carcinoma (one patient with lung adenocarcinoma and the other with rectal cancer).

**Figure 1:**
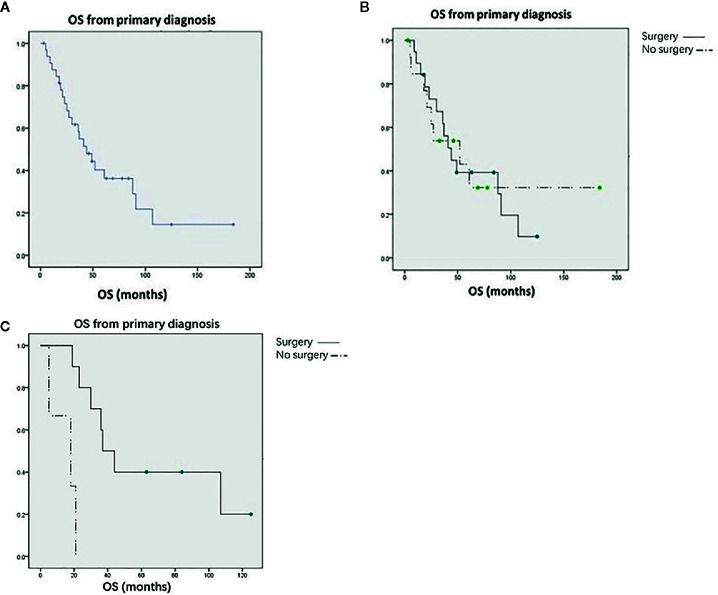
**(A)** Kaplan-Meier curves for overall survival in all patients. **(B)** Kaplan-Meier curves for overall survival in all patients treated with or without renal surgery. **(C)** Kaplan-Meier curves for overall survival in patients without other metastases treated with or without renal surgery.

Thirty-four patients were followed up for an average of 20.8 months (0–139 months). Fifteen patients who underwent surgery and nine patients who did not undergo surgery received systemic therapy, including chemotherapy, targeted therapy, and immunotherapy, after diagnosis. Of the 21 surgical patients, twenty were followed up, and 18 patients recurred and median recurrence free survival of was 7 months (95% CI, 5 to 12). Consequently, on current, two patients (10%) are living with no evidence of disease after surgery. At the end of follow-up, 22 patients had died and 12 were still alive. For the whole study population, the median OS was 44 months (**Figure 1**); the median OS was 44 months for patients who underwent renal surgery and 52 months for patients who did not (**Figure 1**). The Kaplan-Meier survival curve showed that there was no significant difference between these two groups (P = 0. 672). However, for patients without metastasis at other sites, surgery could significantly prolong OS (37 vs. 18 months) (P = 0.001) (**Figure 1**). It is worth noting that a patient with primary thyroid cancer and renal metastasis has survived for more than 11 years after renal surgery. Cox multivariate regression analysis showed that OS only significantly correlated with the age of the patients (P < 0.05), but not with surgery (**Table 2**).

**Table 2:** Factors for OS by multivariate Cox regression analysis.

Variable		OS from primary diagnosis			
		HR	95% CI		P
Age at diagnosis		0.178	0.050	0.64	**0.008**
Gender	Male (Referent)	1.05	1.00	1.12	0.076
	Female				
Primary organ site	Lung (Referent)				
	Others	1.18	0.45	3.13	0.733
Metastasis of other site	No (Referent)				
	Yes	1.60	0.53	4.82	0.403
Symptoms	No (Referent)				
	Yes	1.03	0.34	3.10	0.960
Time to renal metastasis	=<12m (Referent)				
	>12m	1.43	0.54	3.76	0.471
Surgery	Yes (Referent)				
	No	0.90	0.30	2.68	0.849

## Discussion

Considering that the kidneys are highly vascular organs, metastatic infiltration is most likely a result of arterial embolization (5). Currently, there are no data on the incidence of renal metastasis in living patients, and the largest study in this clinical scenario comes from the University of Texas, MD Anderson Cancer Center, which reported 151 cases of renal metastases over a period of about 30 years (4), while our study included 35 patients with renal metastases from two centers.

In terms of primary lesions, this study found that lung cancer and colorectal cancer were the most common primary tumors, which is consistent with previous literature (4). It is worth noting that this study found two cases of renal metastases coexisting with clear cell RCC. These types of cases are also called collision tumors and the incidence rate is very low, with only a small number of cases reported in the past (6–8). The primary kidney tumor can be RCC or renal angiomyolipoma (9). Clear cell RCC is the most common, accounting for 70–75% of all cases, most likely because the high lipid and glycogen content in RCC may provide a favorable environment for metastatic cells from other neoplasms (10, 11). The most common primary foci include the lungs (40–50%), prostate, and thyroid (7). Due to the complexity of the components of collision tumors, there are great differences in imaging findings, but they can be diagnosed based on puncture biopsy or surgical resection pathology (11). The two cases in this study were confirmed by surgical resection pathology. There may be no significant difference in prognosis between collision tumors and simple renal metastases, but this needs to be further confirmed in larger samples.

In addition, metastatic renal tumors are generally small, multiple, bilateral, wedge-shaped, with less exogenous growth and generally located in the renal capsule, while primary renal tumors tend to be single, unilateral, non-wedge-shaped, with an exogenous growth pattern that easily invades the renal capsule (3). However, the accuracy of CT in the diagnosis of renal metastases is not high. In this study, the diagnostic accuracy of enhanced CT was only 45.5%, which was consistent with the 47.5% reported in a previous study (4). Honda et al. reported that the diagnostic accuracy of CT for renal metastatic tumors was 75%, while that for primary renal tumors was 93.2% (3). Zhou et al. reported that clinicians could make a more accurate judgment regarding the presence of renal metastases than imaging doctors (4). The main reason might be that clinicians are more familiar with the medical history and biological behavior of tumors (4). In addition to enhanced CT, it has been reported that PET/CT can detect renal metastases that cannot be detected using CT, and PET/CT can also determine distant metastasis at other sites (12). Some studies have also shown that MRI has some value in the detection of renal metastatic tumors (13).

Metastasis can emerge both early and late in the life of the primary tumor (14), and the aggressiveness and biological behavior of different tumors may lead to different time frames for renal metastasis. Therefore, there is a great difference in the window between the diagnosis of the primary tumor and renal metastasis. Some patients are diagnosed simultaneously, but most metastases appear after the progression of the primary tumor. The longest interval reported in previous studies was 118 months (15). This study found the longest interval was 156 months in a patient with breast cancer.

The significance of surgery for advanced tumors remains controversial. Among them, cytoreduction surgery for sarcoma and colon cancer have been shown to benefit patients (16). For primary renal tumors, previous studies have shown that patients with metastatic RCC can benefit from nephrectomy (17). However, the Carmena study showed that the efficacy of targeted therapy is not inferior to that of targeted therapy plus nephrectomy, which has made urologists more cautious in their choice of tumor-reducing nephrectomy (18). Currently, there remains significant controversy about whether renal metastases require tumor-reducing renal surgery.

Surgical treatment can relieve symptoms,as well as reduce tumor load, and for patients without metastasis at other sites, tumor reduction surgery may achieve a radical effect. On the other hand, renal surgery has some disadvantages. First, patients with metastatic tumors often receive several lines of systemic treatment that can worsen their already poor physical condition, which increases the risk of surgery. Second, such patients often need to receive other systemic treatments after surgery, and renal surgery may delay the time until the provision of systemic treatment, which may lead to disease progression. Abnormal renal function after nephrectomy will also affect the implementation of other treatments.

This study does not show that surgical treatment can prolong OS for all patients with renal metastases. However, we found that for patients without metastasis at other sites, surgery could significantly prolong the OS. Factors related to the favorable outcome were reported to be control of the primary site, confirmed solitary metastatic disease, good performance status, metachronous lesions, and a longer disease-free interval (19).

This study has some limitations. Due to the small sample size, patient selectivity and representativeness are poor, resulting in selective bias and reduced statistical efficiency. Surgical patients, patients with good physical condition, and patients with fewer metastatic sites are generally more likely to receive surgical treatment, and there is a selection bias when comparing OS with patients who did not undergo surgery.

## Conclusions

Renal metastases are rare; however, the most primary focus is the lung. Most patients lack symptoms, and tumors are often found incidentally on imaging examinations. However, imaging examinations may lack specificity. Renal biopsy can be used for diagnosis, but there is a risk of bleeding. Whether surgical treatment of renal metastases can improve the prognosis of patients remains controversial; nonetheless, kidney-targeted intervention could be selected for patients with few metastatic sites and good physical condition.

## Data Availability Statement

The raw data supporting the conclusions of this article will be made available by the authors, without undue reservation.

## Ethics Statement

The studies involving human participants were reviewed and approved by Medical Ethics Committee of Zhejiang Cancer Hospital and Medical Ethics Committee of the Affiliated Hospital of Xuzhou Medical University. The patients/participants provided their written informed consent to participate in this study.

## Author Contributions

SZ was responsible for the conception and design of the study. JC and NQ dealt with the clinical data. JC performed the statistical work and drafted the manuscript. All authors have revised the manuscript. All authors contributed to the article and approved the submitted version.

## Conflict of Interest

The authors declare that the research was conducted in the absence of any commercial or financial relationships that could be construed as a potential conflict of interest.
